# Acute exposure to wood smoke from incomplete combustion - indications of cytotoxicity

**DOI:** 10.1186/s12989-015-0111-7

**Published:** 2015-10-29

**Authors:** Ala Muala, Gregory Rankin, Maria Sehlstedt, Jon Unosson, Jenny A. Bosson, Annelie Behndig, Jamshid Pourazar, Robin Nyström, Esbjörn Pettersson, Christoffer Bergvall, Roger Westerholm, Pasi I. Jalava, Mikko S. Happo, Oskari Uski, Maija-Riitta Hirvonen, Frank J. Kelly, Ian S. Mudway, Anders Blomberg, Christoffer Boman, Thomas Sandström

**Affiliations:** Department of Public Health and Clinical Medicine, Division of Medicine/Respiratory Medicine, Umeå University, Umeå, Sweden; Department of Applied Physics and Electronics, Thermochemical Energy Conversion Laboratory, Umeå University, Umeå, Sweden; Department of Environmental Science and Analytical Chemistry, Arrhenius Laboratory, Stockholm University, Stockholm, Sweden; Department of Environmental Science, University of Eastern Finland, Kuopio, Finland; Environmental Research Group, MRC-PHE Centre for Environment and Health, King’s College London, London, UK

**Keywords:** Air pollution, Biomass, Bronchoscopy, Cytotoxicity, Neutrophils, Lymphocytes, Mast cells

## Abstract

**Background:**

Smoke from combustion of biomass fuels is a major risk factor for respiratory disease, but the underlying mechanisms are poorly understood. The aim of this study was to determine whether exposure to wood smoke from incomplete combustion would elicit airway inflammation in humans.

**Methods:**

Fourteen healthy subjects underwent controlled exposures on two separate occasions to filtered air and wood smoke from incomplete combustion with PM_1_ concentration at 314 μg/m^3^ for 3 h in a chamber. Bronchoscopy with bronchial wash (BW), bronchoalveolar lavage (BAL) and endobronchial mucosal biopsies was performed after 24 h. Differential cell counts and soluble components were analyzed, with biopsies stained for inflammatory markers using immunohistochemistry. In parallel experiments, the toxicity of the particulate matter (PM) generated during the chamber exposures was investigated in vitro using the RAW264.7 macrophage cell line.

**Results:**

Significant reductions in macrophage, neutrophil and lymphocyte numbers were observed in BW (*p* < 0.01, <0.05, <0.05, respectively) following the wood smoke exposure, with a reduction in lymphocytes numbers in BAL fluid (<0.01. This unexpected cellular response was accompanied by decreased levels of sICAM-1, MPO and MMP-9 (*p* < 0.05, <0.05 and <0.01). In contrast, significant increases in submucosal and epithelial CD3+ cells, epithelial CD8+ cells and submucosal mast cells (*p* < 0.01, <0.05, <0.05 and <0.05, respectively), were observed after wood smoke exposure. The in vitro data demonstrated that wood smoke particles generated under these incomplete combustion conditions induced cell death and DNA damage, with only minor inflammatory responses.

**Conclusions:**

Short-term exposure to sooty PAH rich wood smoke did not induce an acute neutrophilic inflammation, a classic hallmark of air pollution exposure in humans. While minor proinflammatory lymphocytic and mast cells effects were observed in the bronchial biopsies, significant reductions in BW and BAL cells and soluble components were noted. This unexpected observation, combined with the in vitro data, suggests that wood smoke particles from incomplete combustion could be potentially cytotoxic. Additional research is required to establish the mechanism of this dramatic reduction in airway leukocytes and to clarify how this acute response contributes to the adverse health effects attributed to wood smoke exposure.

**Trial registration:**

NCT01488500

**Electronic supplementary material:**

The online version of this article (doi:10.1186/s12989-015-0111-7) contains supplementary material, which is available to authorized users.

## Background

It is well known that exposure to ambient air pollution is associated with increased respiratory and cardiovascular morbidity and mortality [1]. While most focus has been on traffic-related air pollution, less attention has been directed towards the impact of smoke from biomass combustion. Source apportionment studies have shown that wood smoke from small-scale combustion for residential heating during winter is a major contributor to PM_2.5_ concentrations in ambient air in large cities around the world [2], with contributions in some European countries (i.e., Sweden, Finland, Germany and Austria) of between 15 and 25 % [3].

Globally, more than 2.4 billion people are estimated to depend on biomass, such as wood, animal dung and crop residues, as a major source of energy for heating and cooking [4]. Furthermore, indoor air pollution has been rated as the third leading risk factor for global disease burden and WHO estimate that exposure to biomass smoke contributed to 3.5 million premature deaths in 2010 [5]. Exposure to wood smoke has been linked to elevated asthma prevalence, increased asthma symptoms in children and adults, as well as higher hospital admissions due to asthma attacks [6–8]. The association between long-term indoor wood smoke exposure and the development and worsening of COPD has been shown to be strong in many epidemiological studies [9, 10] and the risk for COPD development has been estimated to be more than doubled for solid fuel smoke compared with smoke from other types of fuels [11]. Exposure to smoke from combustion of wood and other biomass fuels has also been shown to increase the risk for acute and chronic lower respiratory tract infections, including pneumonias and tuberculosis [7, 12, 13].

The physical and chemical properties of wood smoke particles can differ substantially depending on the combustion conditions and fuel used and it is likely that the physicochemical properties of the smoke particles determine their toxicological properties and health effects [2].

Recently, a series of studies addressed the respiratory and systemic effects of wood smoke using a range of experimental exposures in human subjects. Barregård and colleagues reported varying responses from several wood smoke exposure campaigns. These include increased levels of exhaled malondialdehyde and urinary PGF2α, suggesting enhanced oxidative stress, and some indications of altered coagulability along with increased serum concentrations of club cell protein 16 (CC-16) and amyloid A [14–16]. Other studies have shown no proinflammatory effects in peripheral blood [17, 18]. Ghio et al. reported increased percentages of neutrophils in blood, bronchial wash (BW) and bronchoalveolar lavage (BAL) after exposure to smoldering hardwood [19], We have previously demonstrated a mild oxidative response in BAL fluid, but with no changes in BW or BAL cell numbers in human subjects after experimental exposure to smoke from “low-temperature” incomplete softwood pellet combustion, where the particulate matter PM was dominated by organic matter [20]. In a recent paper [21], based on the same exposure scenario and study population as in the present paper, we investigated the effects of incomplete combustion of birch wood logs, resulting in a sooty polycyclic aromatic hydrocarbon (PAH) rich smoke, representing commonly occurring exposures with chimney stoves. The results from this study confirmed that wood smoke may indeed cause acute cardiovascular effects, demonstrated by increased arterial stiffness, together with reduction in heart rate variability (HRV), which in population-based studies have been strongly linked with adverse health effects [21–23].

The aim of the current study was to further characterize the respiratory effects of exposure to wood smoke from incomplete soot-rich combustion in a typical wood stove. Studies using bronchial mucosal biopsies have previously provided detailed information on the associations between the upregulation of redox-sensitive signaling pathways and the induction of an acute neutrophilic response following diesel exhaust exposure [24–26]. The hypothesis addressed in the present study was that exposure to wood smoke, generated during incomplete combustion conditions, would elicit a similar acute inflammatory response in the airways, as shown after diesel engine exhaust exposure.

Due to the designed differences in combustion conditions, the incomplete combustion scenario, as used in the present and companion paper [21], differs from the previously investigated exposure situation [20], as regards to the properties of the PM emissions. The previously studied wood smoke [20] was generated from softwood pellets during adjusted low-temperature conditions in the burner, with the PM dominated by organic matter (pyrolysis products), whereas the presently used smoke was generated from controlled wood log combustion in a stove under partly incomplete high-burn rate conditions, thus expecting periods of high soot and PAH generation.

## Results

### Wood smoke characterization

The average total PM_1_ concentration in the chamber during the exposures, measured by a TEOM, was 314 μg/m^3^ (range 232–356 μg/m^3^), which was associated with average NO_x_ and CO concentrations of 0.41 ppm and 25 ppm, respectively. Due to the combustion procedure, as described in materials and methods, the concentration of PM and gases in the chamber did vary, although the aerosol residence time in the chamber (~20 min) largely compensated for this. During a typical exposure period, the total particle number concentration in the chamber varied between 1 and 2.5 × 10^5^ particles/cm^3^. The particle number size distribution was bimodal with one peak at 60–70 nm and one peak at 150–200 nm. Previous studies indicate that the 60–70 nm peak consist of alkali salt particles (e.g., K_2_SO_4_ and KCl) and the 150–200 nm peak consists of a soot mode with condensed organic material [27, 28].

The total carbonaceous (TC) PM of the wood smoke was dominated by elemental carbon (EC) and the PM consisted of 38 % soot, 24 % organics and the remainder of ash forming elements (Additional file 1: Table S1 and Additional file 2: Table S2). It is notable that the TC measurements were not done exactly in parallel with the DGI collections, as they demanded different collection times. Thus, exact mass closure is not possible to calculate. The total PAH concentration in the chamber was 1053 ± 648 ng/m^3^, of which 74 % (775 ng/m^3^) was in the particulate phase, see Table 1. The 12 dominating PAH compounds in the PM fraction, accounting for 86 ± 2 % of the total analyzed PAH, were (in descending order); benzo(a)pyrene, benzo(b)fluoranthene, benz(a)anthracene, benzo(e)pyrene, benzo(ghi)perylene, indeno(1,2,3-cd)pyrene, benzo(ghi)fluoranthene, benzo (k)fluoranthene, coronene, pyrene, fluoranthene and perylene. In contrast to diesel exhaust particulates, high molecular weight PAHs (≥228 Da) dominate the PAH profile (85 % in average) of the wood smoke particulate matter. This is expected as the PAHs are purely of pyrogenic origin [29]. The concentrations of all analyzed PAH compounds are given in Additional file 1: Table S1.

**Table 1:** Exposure characteristics

	Unit	Mean ± SD
PM_1_ mass conc. (TEOM)	μg/m^3^	314 ± 38
PM_1_ mass conc. (filter)	μg/m^3^	294 ± 36
CO	ppm	25 ± 6
NOx	ppm	0.41 ± 0.12
EC/TC (elemental/total carbon)		0.72 ± 0.08
Organic fraction of total PM_1_	%	24 ± 8
Soot fraction of total PM_1_	%	38 ± 9.9
PAH-PM associated	μg/m^3^	0.78 ± 0.56
PAH-semi-volatile	μg/m^3^	0.28 ± 0.12

### Immunohistochemistry of the bronchial biopsies

**Fig. 1:**
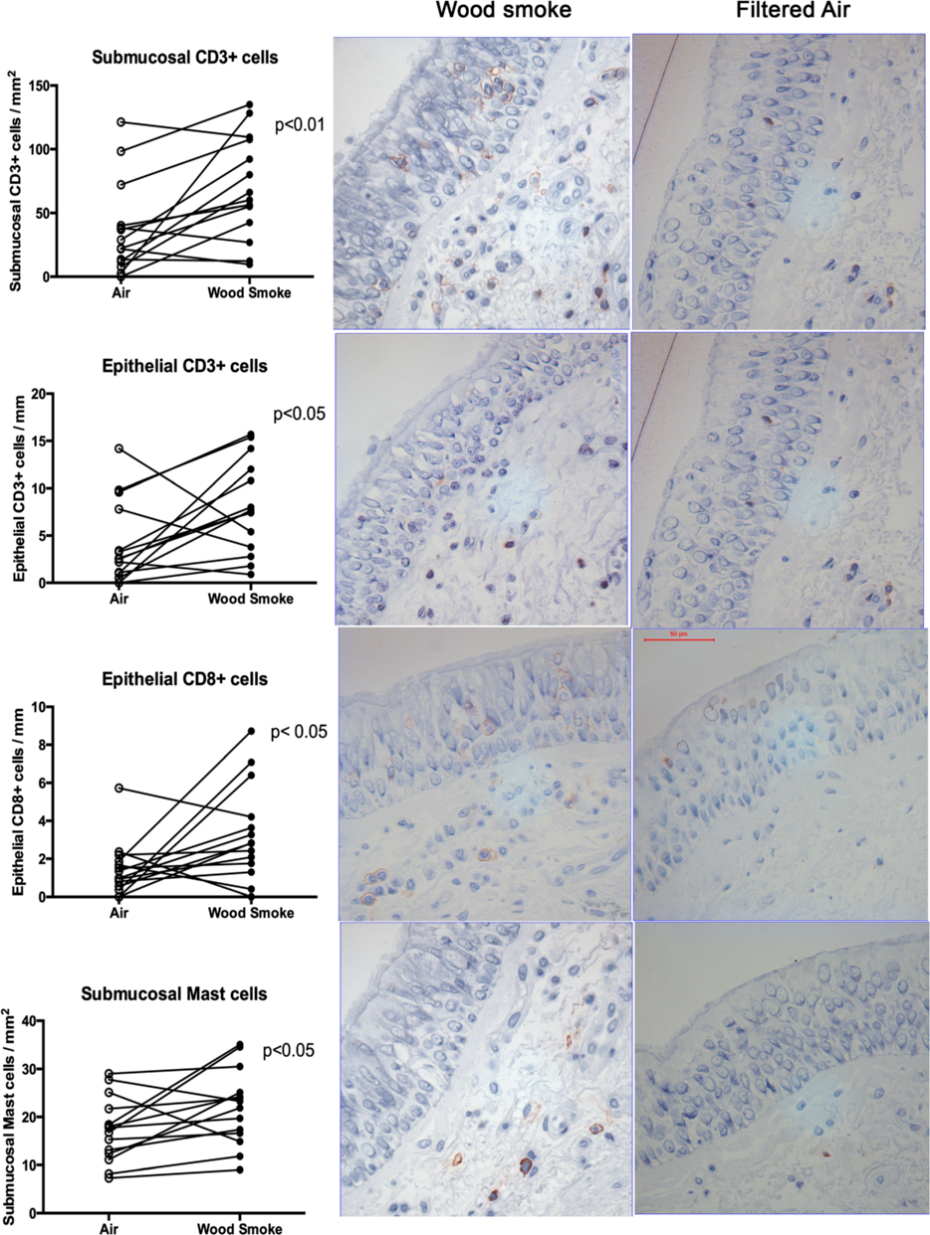
Immunohistochemical staining of bronchial mucosal biopsies. All photos have been taken at the same magnification (×40). Bar represents 50 μm. in order from the top and downwards the four panels demonstrate: a/ A significant increase in submucosal and b/ epithelial CD3+ lymphocytes (*p* < 0.01 and <0.05 respectively), together with c/CD8+ cells in the epithelium (*p* < 0.05) after exposure to wood smoke vs. filtered air. Panel d/ displays that mast cells were significantly increased in the submucosa (*p* < 0.05) after wood smoke exposure. There was no neutrophilic infiltration in the bronchial biopsies (data not shown)

Representative bronchial biopsies were obtained from all 14 subjects after both air and wood smoke exposure. There was a significant increase in submucosal and epithelial CD3+ lymphocytes (*p* < 0.01 and <0.05 respectively), together with CD8+ lymphocytes in the epithelium (*p* < 0.05) after exposure to wood smoke compared to filtered air (Fig. 1). CD4+ lymphocytes were not significantly affected by exposures. Mast cells were increased in the submucosa (*p* < 0.05) after wood smoke exposure (Fig. 1). There were no significant changes in other cell types or adhesion molecule expression after exposure to wood smoke (Additional file 3: Figures S1 and Additional file 4: Figure S2).

### Bronchial wash and bronchoalveolar lavage

**Fig. 2:**
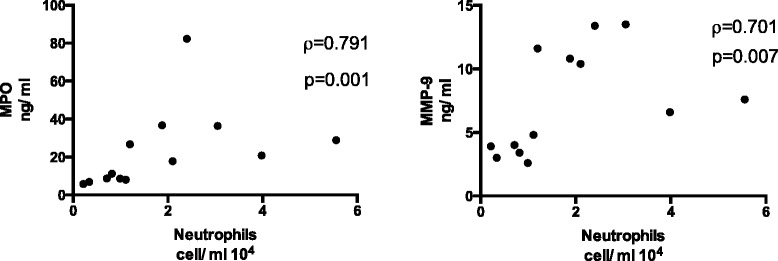
Spearman’s rank correlations between the number concentration of neutrophils in BW and the amount of MPO, as well as MMP-9, obtained after wood smoke exposure

Statistically significant reductions of macrophages, neutrophils and lymphocytes were found in the bronchial wash (BW) (*p* < 0.05- <0.01, Table 2 and Additional file 5: Figure S3) after wood smoke exposure compared to filtered air. Moreover, wood smoke exposure significantly reduced the BW levels of soluble Intercellular Adhesion Molecule-1 (sICAM-1), myeloperoxidase (MPO) and matrix metallopeptidase 9 (MMP-9) (*p* < 0.05- <0.01 (Table 3, Additional file 6: Figure S4). The reductions in neutrophils and decreases in MPO and MMP-9 after wood smoke exposure were strongly correlated, as shown in Fig. 2 (*p* < 0.01, *r* = 0.791 and *p* < 0.01, *r* = 0.70, respectively).

**Table 2:** Bronchial wash and bronchoalveolar lavage cell data from experimental exposure of 13 healthy subjects to filtered air and wood smoke. Data are given as medians with interquartile range

Cells 10^4^/ml	BW		P-value	BAL		P-value
	Air	Wood smoke	Air vs. Wood smoke	Air	Wood smoke	Air vs. Wood smoke
Macrophages	6.1	3.95	0.009	8.50	9.25	0.60
	4.48–12.56	2.60–6.07		8.21–10.05	7.31–12.07	
Neutrophils	3.05	1.19	0.046	0.21	0.15	0.861
	2.42–6.18	0.76–2.72		0.11–0.38	0.05–0.41	
Lymphocytes	0.45	0.26	0.023	2.09	1.62	0.004
	0.21–0.98	0.07–0.31		1.39–2.89	1.16–1.97	
Mast cells	0.009	0.0046	0.061	0.0047	0.01	0.82
	0.002–0.29	0.0018–0.0089		0.00–0.024	0.0024–0.016	
Eosinophils	0.00	0.00	0.345	0.00	0.01	0.441
	0.00–0.01	0.00–0.2		0.00–0.01	0.00–0.03

**Table 3:** Soluble components in bronchial wash and bronchoalveolar lavage from experimental exposure of 13 healthy subjects to filtered air and wood smoke. Data are given as medians with interquartile range

Inflammatory markers	BW		P-value	BAL		P-value
	Air	Wood smoke	Air vs. Wood smoke	Air	Wood smoke	Air vs. Wood smoke
IL6 pg/ml	4.08	3.96	0.646	1.13	1.61	0.515
	2.25–7.25	3.14–5.87		0.79–1.91	0.73–2.15	
sICAM-1 ng/ml	35.20	18.30	0.028	100.45	98.10	0.878
	18.75–45.87	16.72–26.90		53.10–163.22	67.52–126.00	
CC16 ng/ml	582.70	566.60	0.239	328.80	382.60	0.196
	542.45–724.75	556.45–616.10		298.15–438.00	313.05–488.50	
GrzA pg/ml	346.00	242.50	0.221	149.60	113.60	0.753
	178.65–661.95	197.20–266.15		45.35–232.80	62.30–197.20	
MPO ng/ml	26.50	17.80	0.019	2.40	2.10	0.666
	8.05–70.10	8.30–32.65		0.90–5.60	1.05–3.40	
MMP9 ng/ml	13.40	6.60	0.006	0.50	0.70	0.726
	5.55–30.05	3.65–11.20		0.40–1.95	0.20–1.90	
GSx μmol/L	0.88	0.94	0.133	0.63	0.99	0.013
	0.42–1.20	0.58–2.75		0.47–1.02	0.58–1.48	
GSH μmol/L	0.58	0.65	0.173	0.49	0.73	0.064
	0.32–0.95	0.41–2.33		0.27–0.81	0.48–1.13	
GSSG μmol/L	0.10	O.21	0.279	0.04	0.04	0.196
	0.02–0.19	0.05–0.23		0.00–0.13	0.02–0.22

Baseline BW neutrophil values differed slightly from some preceding studies, confirming the importance of internal controls in this kind of study, where every subjects served as his or her control with air and wood smoke exposure in blinded random order.

Due to the significant reduction in cell numbers, markers of cell death (LDH and HMBGP1 as markers of necrosis and active caspase 3 as a marker of apoptosis) were analysed in the BW supernatant. LDH levels were significantly lower after wood smoke exposure compared to filtered air (*p* < 0.05), whereas HMBGP1 levels remained unchanged. The levels of active caspase 3 were below the limits of detection.

In bronchoalveolar lavage (BAL), wood smoke exposure resulted in a significant decrease in the total lymphocyte numbers (Table 2) as well as the total number of CD3+, CD4+, CD8+, CD4 + HLADR+, CD8 + CD314+, CD4 + CD25+ cells (*p* < 0.05-0.01) Table 4, Additional file 7: Figure S5). In contrast, BAL IL-6, sICAM-1, CC16, GrzA, MPO and MMP-9 concentrations were unaffected by the exposures (Table 3). There was a significant increase in total glutathione (GSx) in BAL after exposure to wood smoke (*p* < 0.02), with a trend towards increased reduced glutathione (GSH), (*p* = 0.06) but concentrations of glutathione disulphide (GSSG) remained unchanged (Table 3).

**Table 4:** Flow cytometry data on lymphocyte subsets in bronchoalveolar lavage from experimental exposure of 13 healthy subjects to filtered air and wood smoke. Data are given as medians with interquartile range

	Filtered air	Wood smoke	P-value
			Air vs. Wood smoke
CD3+ ×10^4^/ ml	1.80	1.25	0.006
	1.17–1.34	0.98–1.75	
CD4+ ×10^4^/ ml	1.02	0.74	0.006
	0.78–1.34	0.52–0.98	
CD8+ ×10^4^/ ml	0.60	0.47	0.013
	0.37–0.94	0.24–0.75	
CD16+CD56+ ×10^3^/ml	0.57	0.55	0.055
	0.49–1.18	0.36–0.64	
CD4+CD25+ ×10^3^/ml	0.9	0.68	0.016
	0.77–1.41	0.58–0.97	
CD4+HLADR+ ×10^3^/ ml	1.44	1.16	0.023
	0.96–2.89	0.67–1.81	
CD8+HLADR+ ×10^3^/ml	1.48	1.07	0.087
	0.63–1.82	0.37–1.68	
CD8+CD314+ ×10^3^/ml	2.15	1.32	0.033
	1.18–3.17	0.73–2.56	

### Peripheral blood

Flow cytometry analyses on peripheral blood demonstrated wood smoke exposure led to significant increases in CD16 + CD56+ cells as well as CD4+HLADR+ and CD8+HLADR+ cells (*p* < 0.05-0.01, Table 5), while other cell types were unchanged. No significant changes were seen for IL6, TNF-α, sICAM-1 and CC16 between exposures. (Additional file 8: Table S3).

**Table 5:** Flow cytometry data of peripheral blood lymphocyte subsets after exposure to filtered air and wood smoke in 14 healthy subjects. Data are given as medians with interquartile ranges

Cell × 10^6^/ml	Filtered air	Wood smoke	Filtered air vs. wood smoke
			p-value
CD3+	1.39	1.43	0.064
	1.12–1.98	1.19–2.38	
CD4+	0.78	0.89	0.140
	0.67–1.16	0.60–1.21	
CD8+	0.50	0.55	0.140
	0.35–0.73	0.40–0.83	
CD3-CD16+CD56+	0.12	0.17	0.026
	0.09–0.20.	0.13–0.25	
CD4+HLADR+	0.03	0.04	0.004
	0.02–0.05	0.03–0.05	
CD8+HLADR+	0.08	0.09	0.009
	0.04–0.14	0.05–0.17	

### Lung function test

Lung function parameters were not significantly changed by wood smoke compared with filtered air exposures. (Additional file 9: Table S4).

### FE_NO_

There were no significant differences between changes in FE_NO_ after exposure to wood smoke vs. air. (Additional file 10: Table S5).

### Wood combustion PM induced in vitro toxicity

**Fig. 3:**
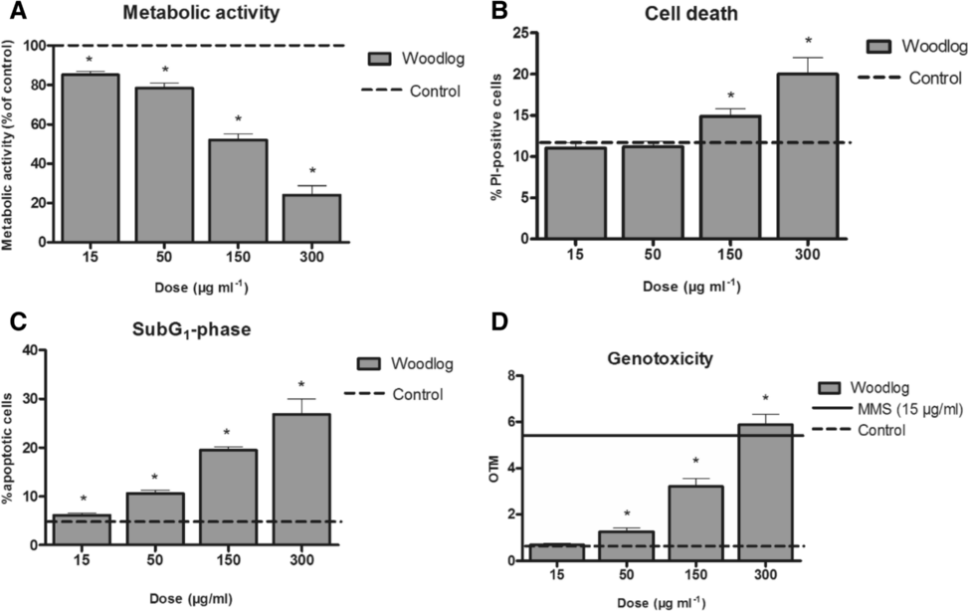
In-vitro data on **a** Cell metabolic activity, **b** percent of propidium iodide (PI) positive cells, **c** percent of subG1 cells and **d** olive tail moment (OTM) in RAW264.7 macrophages after exposure for 24 h to four doses (15, 50, 150 and 300 μg ml^−1^) of cell medium suspended particles collected from a wood log combustion. Each whisker represents the standard error of the mean (SEM). The asterisks (*) indicate a statistically significant difference from control cells (*p* < 0.05, Dunnett’s test or Mann–Whitney U -test)

A concentration-dependent and statistically significant decrease in cellular metabolic activity (MTT, Fig. 3a) and viability (PI, Fig. 3b) was observed after exposure of mouse macrophages to PM_1_ samples from wood log combustion. In contrast, a significant and concentration-dependent increase in the number of subG1 cells was detected in cell cycle analysis (Fig. 3c and Additional file 11: Figure S6A and S6B). Moreover, a concentration-dependent and statistically significant increase in DNA damage was detected when compared to control cells (Fig. 3d). Only a minor PM-induced MIP-2 response was detected in the macrophage cell line, whereas TNF-α concentrations remained at the control level (Additional file 12: Figure S7).

## Discussion

In this study, we examined the pulmonary responses in human subjects exposed to wood smoke from a chimney stove resembling real-world combustion conditions. Under these controlled incomplete combustion conditions, particles were dominated by soot and organic matter. This exposure differed from a previous exposure to wood smoke from incomplete combustion of softwood pellets, as regards to a higher soot fraction and particulate PAH. Overall, the wood smoke in the present study was produced under different fuel, appliance and controlled combustion conditions, that resulted in PM with clearly different chemical properties than the PM in the previous study by Sehlstedt et al. [20]. Based on our observations from previous chamber challenge studies with diesel exhaust, ozone and tobacco smoke, we hypothesized that wood smoke would elicit an acute neutrophilic infiltration of the bronchial tissue [24, 30–33].

Contrary to our hypothesis, we found no evidence of neutrophilic infiltration into the bronchial mucosa, BW or BAL. The lavage fluid response also differed from other experimental air pollution exposure studies, in terms of a decrease in recovered inflammatory cells and soluble components. The basis for the decrease in airway leukocytes remains oblique, but was not associated with an increase in markers indicative of cellular necrosis, apoptosis (LDH, HMBGP1 and active caspase 3) or overt oxidative stress (GSSG). The later result was in agreement with our preceding human exposure study using smoke from a pellet burner [20].

The decrease in the number of airway leukocytes after wood smoke, predominately macrophages, is significant in light of their role in innate immune defense, particularly the clearance of inhaled PM. The reduction in airway leukocytes accompanied with the parallel reduction of the secreted component could potentially increase the susceptibility to airway infections. Indeed, exposure to wood smoke and other biomass smoke has been associated with acute and chronic respiratory infections, including tuberculosis [34, 35]. A meta-analysis of 24 studies demonstrated a two-fold increased risk of pneumonia associated with biomass exposure [13]. Moreover, exposure to wood smoke has been associated with increased mortality by respiratory infections in children [12, 36].

To further clarify this unexpected result, we exposed a macrophage cell line to the wood smoke PM_1_ generated in these exposures, demonstrating a loss of cell viability (mitochondrial activity assessed using the MTT assay) and an increase in DNA damage (dose-dependent OTM response measured by comet assay), associated with a trivial inflammatory response. However, at the highest PM doses (150 and 300 μg ml^−1^) decreased cell viability was detected and thus, the measured OTM value could potentially be due to cell death, rather than PM induced genotoxicity. The macrophage cell line was chosen because the immunological cell response in the lungs demanded further clarification. The RAW 264.7 cells are widely used in air pollution studies as a model for macrophage responses, despite their mouse origin as primary human macrophages are cumbersome to obtain for these kinds of studies. A benefit apart from availability is that RAW 264.7 results can be compared to wide database of previous results, from our lab and others. Moreover, macrophages are a relevant model for unraveling the mechanisms induced by air pollution exposure. The cell line is not aimed for direct interpolation of the results to humans, but rather for revealing the mechanisms of toxicity. Therefore, rather large particle doses were used compared to human exposures in order to generate the differences in toxic responses between wood combustion PM and blank substrate exposures.

The results from the cell exposures are in agreement with other in vitro and in vivo (animal) studies with wood combustion-derived particles, in which the PAH-rich particles have been shown to induce decreased cell metabolic activity and viability [37–39], with an absence of a pronounced inflammatory response [40–42]. Moreover, significant DNA damage in vitro has been associated with the organic carbon (OC) content of PM samples [43–45]. Thus the absence of an overt neutrophilia in the human exposure study might be related to a decreased release of pro-inflammatory mediators due the loss, or impaired function of airway phagocytic cells.

The observation that wood smoke exposure did not elicit an airway neutrophilia was unexpected, however, we cannot totally exclude the possibility that a transient neutrophilic influx occurred earlier than the examined time point. Based on previous studies with other air pollutants, this appears unlikely, especially as no increases in MPO or MMP-9 were found. Ghio et al. reported an increase in the percentage of neutrophils in BW and BAL fluid after a 2-hour exposure of healthy subjects to wood smoke with a particle mass concentration of 500 μg/m^3^, generated from smoldering red oak wood heated on an electric heating element. It is unclear, however, whether the reported airway neutrophilia was due to shifts in the percentages of other inflammatory cell types or whether there was a real expansion in neutrophil numbers [19].

While we observed no evidence of neutrophil influx into the airways post wood smoke exposure, a lymphocytic infiltration of CD8+ cells was found in the bronchial epithelium, together with CD3+ cells and mast cell infiltration in the bronchial mucosa. Again, the increase in cytotoxic CD8+ cells within the epithelium without any significant CD4+ cell infiltration differs from the airway inflammatory cell responses previously observed following exposure to diesel exhaust [24] and tobacco smoke [46].

Mast cell recruitment into the bronchial mucosa has been a common finding after air pollution exposures in human subjects [47–50]. Several earlier studies, which employed BAL alone to evaluate lung responses, reported increased mast cell recovery from the peripheral airspaces after exposure to SO_2_ and NO_2_ [51, 52].

Mast cell infiltration in the bronchial mucosa may be present as early as 6 h after exposure to diesel exhaust and O_3_, and as early as 4 h after SO_2_ and NO_2_. We interpret this as a rapid systemic response, as these cells are released from the bone marrow following stimuli by factors such as stem cell factor, TGF-beta and fractalkine. Particulate matter may induce rapid systemic effects and other not yet identified signals that result in mast cell recruitment from the bone marrow.

Mast cells are often considered in allergy and IgE-mediated reactions but may also be involved in many other immune responses. In the lungs, mast cells have for instance been shown to be present in tissue damage situations and there is debate whether they are mainly involved in enhancing damage or in the restoration phase [53]. It appears that mast cells have capability for both. The mast cell pro-inflammatory potential to release cytokines, histamine and proteases is well known and they may orchestrate inflammatory cascades and cell recruitment. Mast cells may also be important in the response to cell injury by several pathways including IL-33 secreted by damaged or necrotic cells [54].

With regard to the systemic effects of wood smoke exposure, we have recently reported from the present exposure study that inhalation of the investigated sooty wood smoke caused an immediate increase in central arterial stiffness with reduced heart rate variability [21]. This type of response has been associated with adverse long-term outcome in regards to morbidity and mortality [22, 55, 56]. This cardiovascular response occurred in the absence of overt systemic neutrophilia, or increase in circulating pro-inflammatory mediators. This is in line with observations from earlier humans challenge studies using various types of wood smoke, which failed to show increased levels of IL-6 and TNF-alpha, with only minor or no indications of oxidative stress and DNA-damage [17, 18]. We did observe a 1.5 fold increase in blood CD16/CD56 NK cells and increased expression of the activation marker HLA-DR on CD4 and CD8 cells. This was consistent with findings by Dutta and coworkers in India, who used flow cytometry to determine immune markers in blood of women exposed to biomass smoke. These investigators also reported changes in CD4, CD8 and CD19 cells, in contrast to our data, which may be related to alterations in systemic immunity by long-term biomass exposure [57]. This is supported by results from a cross sectional study in which, wood smoke exposure caused high gene expression of mediators linked to airway inflammation and remodeling [58].

## Conclusions

Short-term exposure to soot- and PAH-rich wood smoke did not induce a neutrophilic airway inflammation, which has been a hallmark of acute air pollution exposures in humans. While the current study demonstrated only minor proinflammatory lymphocytic and mast cell effects in the bronchial biopsies, there were unexpected reductions in BW and BAL cell numbers and soluble inflammatory markers. These findings are supported, in part, by in vitro data from the present and other studies which indicate that PAH-rich wood smoke particles from incomplete combustion have the capacity to cause cellular dysfunction and DNA damage. Further research is needed to determine the precise role of these events in relationship to the adverse health effects attributed to wood smoke exposure.

## Methods

### Subjects

Fourteen healthy volunteers (mean age 26, range 21–35 year, 8 males, 6 females, all never smokers) were included. All subjects underwent a physical examination, baseline blood count and renal function assessment, spirometry (FEV_1_, VC and FEV_1_/VC) and 12 lead electrocardiogram prior to inclusion. All were free of airway infection for at least 6 weeks prior to participation. The study was approved by the regional ethical review board and performed in accordance with the declaration of Helsinki. All subjects gave their written informed consent.

### Study design

The study was performed in a randomized, double blind, crossover fashion with each subject being exposed on two occasions to air and wood smoke in an exposure chamber at Thermochemical Energy Conversion Laboratory at Umeå University. The chamber is made of stainless steel, has a volume of a 15 m^3^ and an air exchange rate of around three times per hour and has been previously described [21]. The mean PM concentration of wood smoke was 314 μg/m^3^. The exposures lasted for three hours, during which the subject performed intermittent exercise on a bicycle ergometer, alternate with rest at 15-minute intervals, to achieve an average minute ventilation of 20 L/min/m^2^ body surface. During the exposures, symptoms were recorded according to the modified Borg scale, as described previously [59].

### Wood smoke generation

A common Nordic chimney wood stove was used to generate wood smoke under incomplete combustion conditions. Birch wood logs with a moisture content of 16–18 % were inserted every 5–15 min to maintain a high burn rate with repeated air-starved conditions. The exposure conditions, measurements and characteristics have been described in detail in a companion paper in this journal, which reported on the cardiovascular effects of the same wood smoke exposure and subjects as in the present study [21].

PM_1_ samples, for the toxicological analyses in vitro, were collected on polytetrafluoroethylene (PTFE) substrates from the chamber exhaust air with a Dekati gravimetric impactor (DGI, Dekati Ltd., Finland). The handling of the sampling substrates prior and after collection is described earlier in detail by Jalava et al. 2012.

### Lung function test

Lung function tests were performed before as well as 0 and 24 h after exposure (pre-bronchoscopy) using a spirometer (Jaeger MSC spirometer, Germany). The tests were performed according to the guidelines of the American Thoracic Society [60].

### Fraction of exhaled nitric oxide

Fraction of exhaled nitric oxide (FeNO) at the flow rates of 50 ml/s and 10 ml/s were measured before as well as 0 and after 24 h after each exposure by using a chemiluminescence analyser (NiOX; Aerocrine AB, Stockholm, Sweden).

### Blood samples

Blood samples were obtained at baseline, 24 and 44 h after exposure. The samples were centrifuged at 3,000 × *g* for 30 min at 4 °C before plasma was removed and frozen at −80 °C for further analysis. Plasma samples were analysed for markers of acute inflammation: Interleukin 6 (IL-6), tumor necrosis factor-alpha (TNF-α), soluble Intercellular Adhesion Molecule-1 (sICAM-1) and club cell secretory protein 16 (CC16, formerly called clara cell protein 16) using DuoSet ELISA kits (R&D Systems, Abingdon, UK), according to the manufacturer’s instructions.

### Bronchoscopy

Bronchoscopy was performed 24 h after each exposure using a flexible video bronchoscope (Olympus BF IT200, Tokyo, Japan), as previously described [24, 31]. The subjects received topical anesthesia with Lidocain in pharynx, epipharynx and within the bronchial tree. Bronchial wash (BW), 2 × 20ml, and bronchoalveolar lavage (BAL), 3 × 60 ml, were collected. The obtained aspirates were collected in separate containers and placed in ice before being filtered through a nylon filter (pore diameter of 100 μm) and centrifuged at 400 × *g* for 15 min. Supernatants were frozen at −80 °C for later analyses for; IL-6, club cell protein-16 (CC-16), Granzyme A (GrzA), soluble-intracellular adhesion molecule-1 (s-ICAM-1), matrix metallopeptidase 9 (MMP-9), myeloperoxidase (MPO) (R&D Systems, Abingdon, UK), high-mobility group box protein 1 (HMGB1) (IBL International, Hamburg, Germany), human active-caspase 3 (Invitrogen, Camarillo, USA), and lactate dehydrogenase (LDH) (Roche, Basel, Switzerland), performed according to the manufacturer’s instructions. Endobronchial mucosal biopsies were taken either from the anterior part of the main carina and the subcarinae of the third and fourth generation airway of the right side or from the posterior part of the main carina and the subcarinae on the left side. The order of left/right bronchial biopsy sampling was randomized, with contralateral biopsies taken at the second bronchoscopy.

### Immunohistochemical analysis

Endobronchial mucosal biopsies obtained during bronchoscopy were processed and embedded in glycol methacrylate resin (Polyscience; Northampton, England), as previously described [24]. 2 μm thick sections were cut and immunostained with monoclonal mouse antibodies for the following markers: Neutrophil elastase; mast cell tryptase (Dako); CD3 (Biolegend); CD4 (Biolegend); CD8 (Dako). The immunostaining procedure followed has been described previously [24]. Briefly, endogenous peroxidases were inhibited using a sodium azide and hydrogen peroxide solution and nonspecific antibody binding was blocked using undiluted culture medium (Sigma; St Louis, Missouri). Mouse anti-human antibodies were applied and incubated overnight at room temperature. After washing with Tris-buffered saline, the biotinylated rabbit anti-mouse secondary antibody (IgG F[ab’]_2_; Dako) was applied and incubated for 2 h. After further washing a streptavidin-biotin horseradish peroxidase complex (Vector Laboratories) was added and incubated for 2 h. The sections were then visualized using 3-amino-9-ethylcarbazole (AEC) (Vector Laboratories, Land) and counterstained with Mayer’s haematoxylin. Positively stained nucleated cells were counted within the submucosa, excluding smooth muscle and glands, and in intact epithelium. Counts were corrected for submucosal area and epithelial length using the program LeicaQWin V3 (Leica Q500IW; Leica, Cambridge, UK). Activated blood vessels were expressed as the ratio of p-selectin and ICAM-1 positive vessels to the pan-endothelial marker EN4-positive vessels.

### Flow cytometry

Flow cytometry was used to analyze lymphocyte subsets in BAL and in the peripheral blood as previously described [61].

### Antioxidant analysis

Reduced glutathione (GSH), oxidized glutathione (GSSG) and total glutathione (GSx) were measured in bronchial wash and BAL fluid returns as described previously [62].

### Toxicological analyses in vitro

#### Cell culture

Mouse macrophage cells (RAW264.7, ATCC, USA) were grown in RPMI 1640 medium supplemented with 10 % heat inactivated fetal bovine serum, 2 mM L-glutamine, and 100 U mL^−1^ penicillin − streptomycin in a humid atmosphere of 5 % CO2 (+37 °C). On the day before the experiments, the cell suspension at a concentration of 5 × 10^5^ cells mL^−1^ (2 mL/well) was dispensed into 6-well plates (total cell count for the well is 1x10^6^, Corning Inc., USA). Fresh culture medium (2 mL/well) was changed 1 h before exposure of the cells to particles or their controls.

#### Cell exposure

Half an hour before the exposure, emission particles were suspended into DMSO (20 μL mg^−1^, Merck KGaA, Germany). After that, pathogen-free water (W1503, Sigma-Aldrich Corp., USA) was added to gain a PM concentration of 5 mg mL^−1^. This suspension was sonicated in an ultrasonic water bath (FinnSonic m03, FinnSonic Ltd., Finland) for 30 min below +35 °C to gain homogenous mixture. Mouse RAW264.7 macrophages were exposed for 24 h to four doses (15, 50, 150, and 300 μg mL^−1^) of emission particles from wood log combustion. Exposures of the cells to the particulate samples were made at least in three independent experiments. All in vitro experiments contained diesel PM (dose 150 μg ml^−1^) [63] and blank substrate (dose 150 μg ml^−1^) controls. Moreover water (dose 1.7 mM) and DMSO (dose 41.1 μM) served as vehicle controls. Blank sample virtual mass was calculated to be equivalent for PM containing filters average mass.

#### Toxicological analyses

After the 24 h exposure on 6 well plates, the macrophages were scraped from the wells and a sample for MTT-test was taken (200 μl). The rest of the cell suspension was centrifuged (6.081 g, +4 °C, Heraeus Biofuge Fresco, Land) to separate the cells and particles from the cell culture medium. The supernatant was stored at −80 °C for the analysis of inflammatory mediators. The cells were suspended into 1 mL of PBS (Gibco, UK), and half of them were used in a propidium iodide exclusion assay, and the other half was fixed with ethanol (70 % v/v, Altia, Finland) and stored at +4 °C for DNA content analysis with flow cytometry (CyAn ADP, Beckman Coulter Inc., USA). The other duplicate part of the cells was used without delay in the Comet assay. Inflammatory mediators were also analyzed from those cells culture medium. Moreover, 200 μl aliquot of scraped cell suspension was used for MTT test.

#### Cytotoxicity

##### MTT test

The mitochondrial activity of the macrophages was analysed with the MTT-test on 96-well plates, which was used for calculating the proportion of the viable cells. The absorbance was detected with the spectrophotometric plate reader and the viability was calculated as a percentage from corresponding readings of the control cells.

##### Cell cycle analysis

The cellular DNA content was analyzed from ethanol fixed cells (12,000 cells) by propidium iodide (PI) staining in a flow cytometer (CyAnTM ADP, Beckman Coulter, CA, USA). With this method, cells in the different phases of the cell cycle can be identified. Cells in subG1 phase, which indicates apoptotisis, can be identified as they contain fragmented DNA [64]. In the cell cycle analysis, the fixed cells were treated 0.15 mg ml^−1^ of ribonuclease A and stained with PI (Sigma Aldrich Corp.) at a final concentration of 8 μg ml^−1^. Etoposide (dose 2 μM) was used as a positive control in cell cycle analyses.

##### PI exclusion

The total amount of PI positive cells was assayed from freshly scraped cells using flow cytometry (CyAnTM ADP, Beckman Coulter, CA, USA). To separate cells and culture medium, the cell suspensions were centrifuged (370 × g, 5 min) and the cell pellet was resuspended in phosphate buffered saline (PBS). Cells were washed once with PBS before staining with PI (0.5 ml PBS, 1 μg/ml PI) Thereafter, cells were immediately analysed using excitation at 488 nm and emission filter 613 ± 20 nm (channel FL 3). A total of 12,000 cells were analysed for their PI content using Summit software version 4.3 (Beckman Coulter, CA, USA). PI positivity indicated compromised cell membrane.

##### Genotoxicity

DNA damage was detected in the alkaline Single cell gel/Comet assay done in three independent experiments. In the comet assay, DNA strand breaks and breaks associated with incomplete excision repair sites (alkaline labile) can be detected, and therefore the potential genotoxicity of sample can be estimated. The nuclei were analyzed in ethidium bromide-stained cells (100 cells per dose) using an image analysis system (Comet assay IV, Perceptive Instruments Ltd., Suffolk, UK). The Olive tail moment [(tail mean – head mean) x tail%DNA/100)] was the parameter used in the statistical analysis. Methyl methanesulfonate (MMS) (dose 15 μg/ml) and benzo [a] pyrene (dose 240 μM) were used as positive controls in the analyses of genotoxicity.

#### Cytokine analysis

Tumor necrosis factor alpha (TNF-α) and macrophage inflammatory protein-2 (MIP-2) concentrations were analyzed from cell culture medium. Cytokine analysis was made with commercially available enzyme-linked immunosorbent assay (ELISA) kits (R&D Systems, Minneapolis, MN, USA) according to the manufacturer’s instructions. Lipopolysaccharide (dose 0.01 μg/ml) was used as positive controls in the analyses of cytokines.

## Statistics

Wilcoxon signed-rank test was used for comparison of BW, BAL, flow cytometry and immunohistochemical data. Following confirmation of normality by the Shapiro-Wilk test, paired sample *T*-test was used for lung function and FENO data. Correlations between wood smoke induced effects in BW were analyzed using the Spearman rank order correlation test.

A linear mixed-effect model was used to analyze the impact of wood smoke on systemic inflammatory markers. Subjects were selected as random effect, and exposure, time, order of exposures and interaction between exposure and time as fixed effects. Lung function and FeNO data, which were normally distributed, are presented as mean with ± SD, and non-normally distributed data as medians with interquartile range.

The measured toxicological responses of PM exposed cells were compared to the blank substrate exposed cells. Levene’s test for equality of variances was used for all the samples before analyzing the data with ANOVA. ANOVA and Dunnett’s post hoc test was used when results from the MTT-test (*n* = 6) and cytokines (*n* = 6) was analyzed. The results from the OTM analyses, PI-exclusion assay, and cell cycle analyses were analyzed in a nonparametric Mann–Whitney *U* test (*n* = 3). Differences were considered to be statistically significant at *p* < 0.05.

Data were analyzed using SPSS, version 20 for Macintosh (IBM® SPSS® Statistics 20, Chicago, IL, USA) and GraphPad Prism (GraphPad software version 6 for Macintosh, San Diego, CA, USA).

## Additional files

Additional file 1: Table S1. Concentrations (ng/m3) of specific PAH compounds in the semi-volatile phase (semi-vol) and particulate phase (PM) of the wood smoke (WS) (*N* = 10) data given as mean with SD, and air (*N* = 2) data given as mean. (DOCX 89 kb) Additional file 2: Table S2. Ash forming elements and anions analyzed from the wood log combustion particulate sample with ICP-MSa and ICb. (DOCX 45 kb) Additional file 3: Figure S1. Scatterplot of inflammatory cells in the bronchial submucosa after air and wood smoke exposure. (PDF 50 kb) Additional file 4: Figure S2. Scatterplot of inflammatory cells in the bronchial epithelium after air and wood smoke exposure. (PDF 49 kb) Additional file 5: Figure S3. Scatterplot of inflammatory cells in bronchial wash after air and wood smoke exposure. (PDF 45 kb) Additional file 6: Figure S4. Scatterplot of soluble components in bronchial wash after air and wood smoke exposure. (PDF 50 kb) Additional file 7: Figure S5. Scatterplot of inflammatory cells in bronchoalveolar lavage after air and wood smoke exposure. (PDF 45 kb) Additional file 8: Table S3. Soluble components in the peripheral blood from experimental exposure of 14 healthy subjects to filtered air and wood smoke. Samples were collected before (pre), at 24 h and 44 h after exposure, to air and wood smoke. Data are given as medians with interquartile range. (DOCX 62 kb) Additional file 9: Table S4. Lung function data from 14 subjects before (pre), immediately after (post) and 24 h after experimental exposure to filtered air and wood smoke. Data are given as mean with ± SD. (DOCX 46 kb) Additional file 10: Table S5. FENO data from 14 healthy subjects sampled before (pre), immediately after (post) and 24 h after experimental exposure to filtered air and wood smoke. Data are given as mean with ± SD. (DOCX 45 kb) Additional file 11: Figure S6A. The percentages of mouse RAW264.7 macrophages in the different phases of the cell cycle (SubG1, G1 and S-G2/M) after exposure to different concentrations (15, 50, 150 and 300 μg ml-1) of emission particles from wood log combustion. Asterisks indicate statistical significance compared to control (*p* < 0.05) analyzed by non-parametric KruskaleWallis test. **Figure S6B.** The percentages of mouse RAW264.7 macrophages in the different phases of the cell cycle (SubG1, G1 and S-G2/M) after exposure to etoposide and blank substrata. (PDF 154 kb) Additional file 12: Figure S7. Inflammation mediators tumor necrosis factor alpha (TNF-α) (A) and macrophage inflammatory protein 2 (MIP-2) (B) concentration in cell culture medium after RAW264.7 macrophages were exposure to four doses (15, 50, 150 and 300 μg ml-1) at 24 h of cell medium suspended particles emitted from a wood log combustion or lipopolysaccharide (LPS). The asterisks (*) indicate a statistically significant difference from control cells (*p* < 0.05, Dunnett’s test). (PDF 147 kb)

## Abbreviations

CC16: Club cell protein 16 (formerly called Clara Cell protein 16)
CD4: Cluster of differentiation 4
CD8: Cluster of differentiation 8
EC: Elemental carbon
FeNO: Fraction of exhaled nitric oxide
FEV_1_: Forced expiratory volume in one second
GOLD: The global initiative for chronic obstructive lung disease
GrzA: Granzyme A
GSH: Reduced glutathione
GSSG: Oxidized glutathione
GSx: Total glutathione
HMGB1: High-mobility group box protein 1
ICAM-1: Intercellular adhesion molecule 1
IL-6: Interleukine-6
IL-8: Interleukine-8
IL-13: Interleukine-13
MIP-2: Macrophage inflammatory protein 2
MMP-9: Matrix MetalloPeptidase-9
MPO: Myeloperoxidase
NF-kB: Nuclear factor-kappa B
NK: Natural killer cells
OC: Organic carbon
PAH_s_: Polycyclic aromatic hydrocarbons
ROS: Reactive oxygen species
RTLF: Respiratory tract lining fluid
SDMA: Symmetric dimethylarginine
SNP: Sodium nitroprusside
TC: Total carbon
TEOM: Tapered elemental oscillating microbalance
TNF-α: Tumor necrosis factor alpha
