# Supplementary material for: Acute exposure to wood smoke from incomplete combustion - indications of cytotoxicity
Source: Part Fibre Toxicol. 2015 Oct 29;12:33. doi: 10.1186/s12989-015-0111-7 (PMC4625445; doi:10.1186/s12989-015-0111-7)
Supplement: Additional file 1: Table S1. — Concentrations (ng/m3) of specific PAH compounds in the semi-volatile phase (semi-vol) and particulate phase (PM) of the wood smoke (WS) (N = 10) data given as mean with SD, and air (N = 2) data given as mean. (DOCX 89 kb) [file 12989_2015_111_MOESM1_ESM.docx]

**Table s1**: Concentrations (ng/m^3^) of specific PAH compounds in the semi-volatile phase (semi-vol) and particulate phase (PM) of the wood smoke (WS) (N=10) data given as mean with SD, and air (N=2) data given as mean.

|  | **WS (semi-vol)** | **SD** | **Air (semi-vol)** | **WS (PM)** | **SD** | **Air (PM)** |
| --- | --- | --- | --- | --- | --- | --- |
| Phenanthrene | 106 | 49.0 | 68.1 | 3.61 | 2.21 | 1.82 |
| Anthracene | 9.99 | 4.10 | 2.18 | 1.19 | 0.781 | 0.413 |
| 3-Methylphenanthrene | 4.93 | 1.94 | 2.45 | 0.264 | 0.193 | 0.200 |
| 2-Methylphenanthrene | 5.35 | 1.98 | 2.56 | 0.342 | 0.253 | 0.217 |
| 2-Methylanthracene | 0.978 | 0.467 | 0.168 | 0.101 | 0.067 | 0.069 |
| 9-Methylphenanthrene | 2.81 | 1.15 | 1.40 | 0.183 | 0.140 | 0.102 |
| 1-Methylphenanthrene | 4.11 | 1.75 | 1.90 | 0.331 | 0.261 | 0.198 |
| 4H-Cyclopenta(def)phenanthrene | 6.34 | 3.16 | 1.96 | 0.488 | 0.378 | 0.254 |
| 2-Phenylnaphthalene | 6.89 | 3.60 | 4.41 | 0.932 | 0.801 | 0.444 |
| 3,6-Dimethylphenanthrene | 0.416 | 0.174 | 0.192 | nd | nd | 0.018 |
| 3,9-Dimethylphenanthrene | 1.62 | 0.599 | 0.844 | 0.133 | 0.123 | 0.060 |
| Fluoranthene | 47.8 | 30.4 | 21.8 | 17.8 | 19.8 | 2.62 |
| Pyrene | 57.7 | 35.6 | 19.1 | 23.2 | 25.4 | 2.54 |
| 1-Methylfluoranthene | 3.38 | 1.59 | 1.10 | 6.30 | 6.65 | 0.647 |
| Benz(a)fluorene | 2.80 | 1.41 | 0.951 | 3.15 | 2.74 | 0.273 |
| Benz(b)fluorene | 1.41 | 0.762 | 0.478 | 1.56 | 1.20 | 0.147 |
| 2-Methylpyrene | 1.21 | 0.690 | 0.419 | 3.02 | 3.07 | 0.057 |
| 4-Methylpyrene | 1.68 | 0.733 | 0.484 | 4.74 | 4.47 | 0.272 |
| 1-Methylpyrene | 2.47 | 1.26 | 0.677 | 5.75 | 5.28 | 0.292 |
| Benzo(ghi)fluoranthene | 2.15 | 1.63 | 0.839 | 46.4 | 45.4 | 1.20 |
| Benzo(c)phenanthrene | 6.50 | 7.48 | 0.908 | 14.2 | 14.1 | 0.427 |
| Benzo(b)naphto(1,2-d)thiophene | 0.006 | 0.004 | 0.010 | nd | nd | nd |
| Benz(a)anthracene | 1.32 | 2.03 | 0.511 | 77.4 | 69.2 | 1.24 |
| 3-Methylchrysene | 0.037 | 0.027 | nd | 3.75 | 1.98 | 0.014 |
| 2-Methylchrysene | 0.071 | 0.085 | 0.013 | 7.61 | 4.51 | 0.149 |
| 6-Methylchrysene | 0.167 | 0.197 | 0.038 | 5.47 | 3.16 | 0.102 |
| 1-Methylchrysene | 0.060 | 0.071 | 0.007 | 8.07 | 3.77 | 0.145 |
| Benzo(b)fluoranthene | 0.591 | 0.589 | 0.081 | 99.1 | 71.7 | 0.615 |
| Benzo(k)fluoranthene | 0.423 | 0.558 | 0.030 | 47.4 | 34.4 | 0.348 |
| Benzo(e)pyrene | 0.292 | 0.304 | 0.036 | 76.3 | 51.9 | 0.443 |
| Benzo(a)pyrene | 0.662 | 0.847 | 0.036 | 108 | 70.8 | 0.586 |
| Perylene | 0.341 | nd | nd | 16.0 | 10.1 | 0.177 |
| Indeno(1,2,3-cd)fluoranthene | 0.375 | 0.176 | nd | 7.61 | 5.57 | 0.042 |
| Indeno(1,2,3-cd)pyrene | 0.223 | 0.254 | nd | 61.7 | 41.6 | 0.407 |
| Dibenz(a,h)anthracene | 0.059 | 0.055 | nd | 8.57 | 5.74 | 0.066 |
| Picene | 0.056 | nd | 0.016 | 9.26 | 5.78 | 0.072 |
| Benzo(ghi)perylene | 0.185 | 0.185 | 0.021 | 69.4 | 44.1 | 0.225 |
| Dibenzo(a,l)pyrene | nd | nd | nd | 1.17 | 0.622 | nd |
| Dibenzo(a,e)pyrene | nd | nd | nd | 6.04 | 3.41 | nd |
| Coronene | 0.123 | 0.010 | 0.027 | 26.3 | 16.9 | 0.115 |
| Dibenzo(a,i)pyrene | nd | nd | nd | 1.54 | 0.879 | nd |
| Dibenzo(a,h)pyrene | nd | nd | nd | 0.632 | 0.287 | nd |
| **SUM (ng/m3)** | **277** | **121** | **134** | **775** | **562** | **17.0** |
