# Supplementary material for: Acute exposure to wood smoke from incomplete combustion - indications of cytotoxicity
Source: Part Fibre Toxicol. 2015 Oct 29;12:33. doi: 10.1186/s12989-015-0111-7 (PMC4625445; doi:10.1186/s12989-015-0111-7)
Supplement: Additional file 2: Table S2. — Ash forming elements and anions analyzed from the wood log combustion particulate sample with ICP-MSa and ICb. (DOCX 45 kb) [file 12989_2015_111_MOESM2_ESM.docx]

**Table S2.** Ash forming elements and anions analyzed from the wood log combustion particulate sample with ICP-MS^a^ and IC^b^.

|  | |
| --- | --- |
| Component | [ng/mg] |
| Cl^−^ | 8970 |
| Ca | 5600 |
| Cd | 38 |
| Cr | 140 |
| Cu | 246 |
| F^-^ | BDL |
| Fe | BDL |
| K | 16140 |
| Mg | 1794 |
| Mn | BDL |
| Na | 4840 |
| Ni | BDL |
| NO_3_^-^ | 5350 |
| Pb | 158 |
| Rb | 184 |
| Si | 132200 |
| SO_4_^2-^ | 7880 |
| Zn | 5160 |

BDL = below detection limit
^a^Mass of multiple elements (Ca, Cd, Cr, Cu, Fe, K, Mg, Mn, Mo, Na, Ni, Pb, Si, Sr, and Zn) were analyzed using inductively coupled plasma mass spectrometer (ICP-MS PerkinElmer Elan 6000)
^b^Anions (Cl^−^, Br^−^, F−, NO_3_^−^ and SO_4_ ^2−^) were determined using chromatography (IC, Dionex DX-120)
Sample preparation has been described in detail by Uski et al. 2015.
