# Supplementary figures and images for: Acute exposure to wood smoke from incomplete combustion - indications of cytotoxicity
Source: Part Fibre Toxicol. 2015 Oct 29;12:33. doi: 10.1186/s12989-015-0111-7 (PMC4625445; doi:10.1186/s12989-015-0111-7)

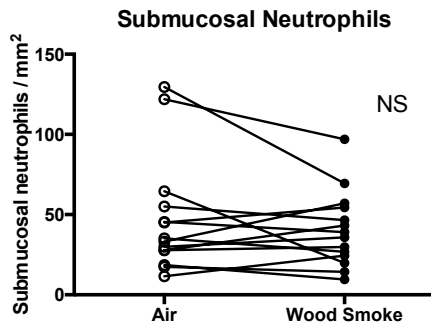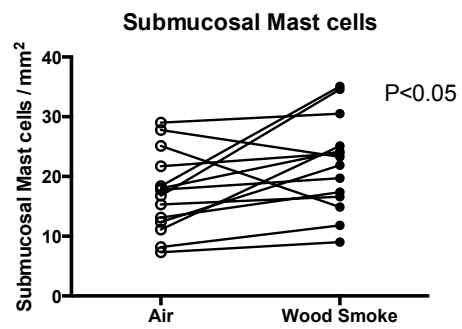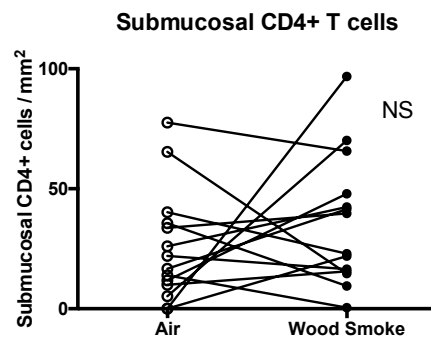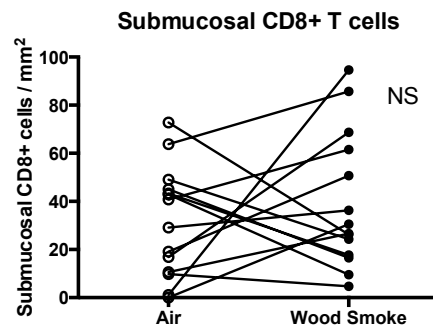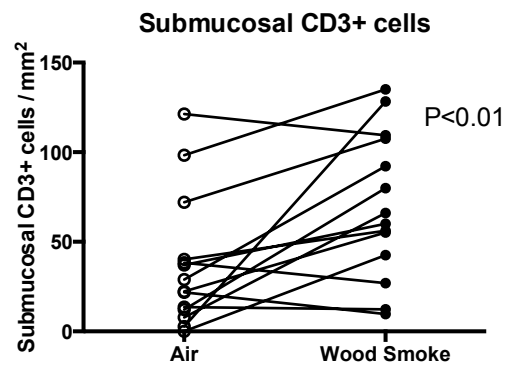

Supplement: Additional file 3: Figure S1. — Scatterplot of inflammatory cells in the bronchial submucosa after air and wood smoke exposure. (PDF 50 kb) [file 12989_2015_111_MOESM3_ESM.pdf]

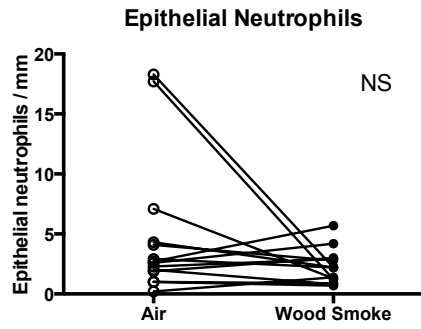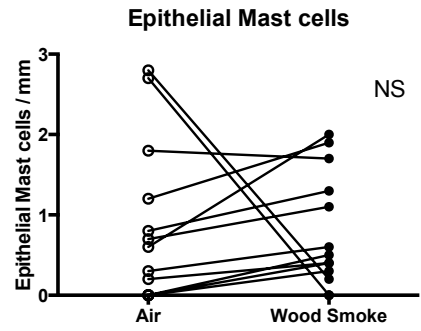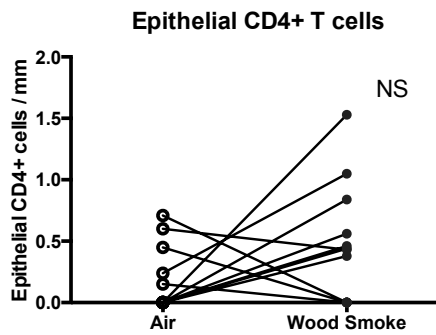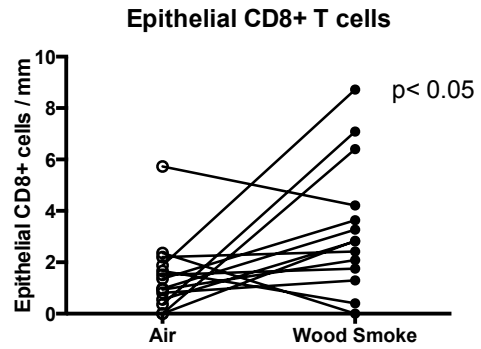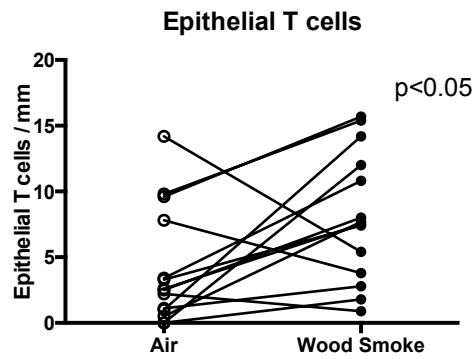

Supplement: Additional file 4: Figure S2. — Scatterplot of inflammatory cells in the bronchial epithelium after air and wood smoke exposure. (PDF 49 kb) [file 12989_2015_111_MOESM4_ESM.pdf]

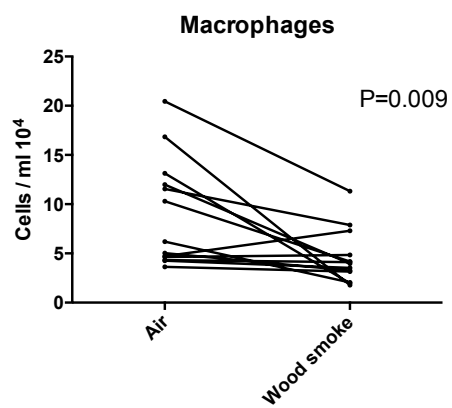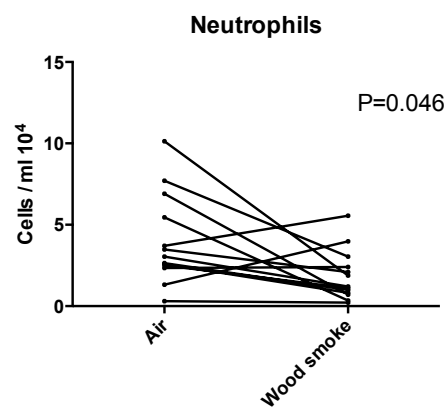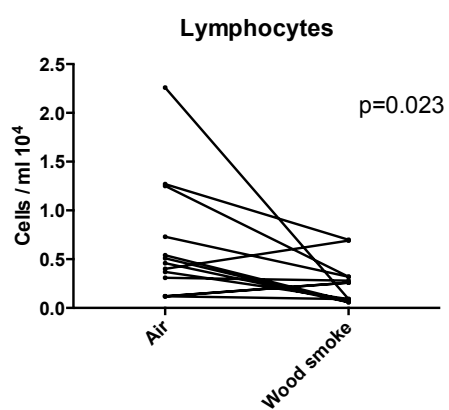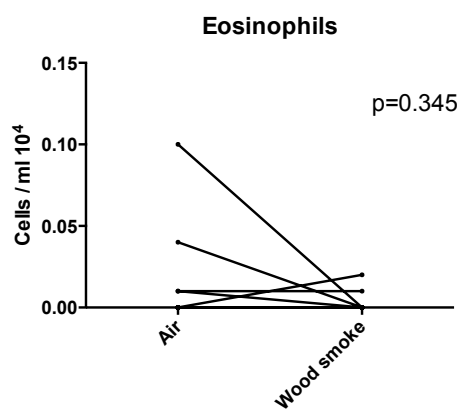

Supplement: Additional file 5: Figure S3. — Scatterplot of inflammatory cells in bronchial wash after air and wood smoke exposure. (PDF 45 kb) [file 12989_2015_111_MOESM5_ESM.pdf]

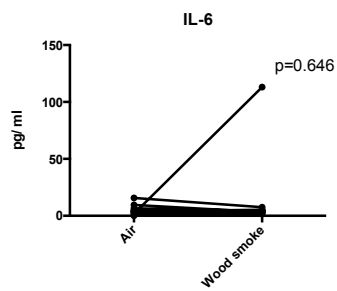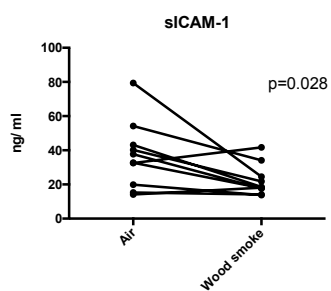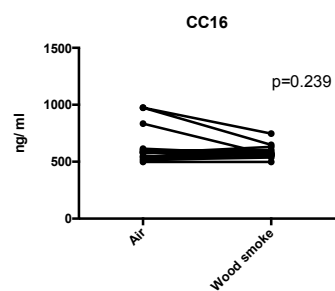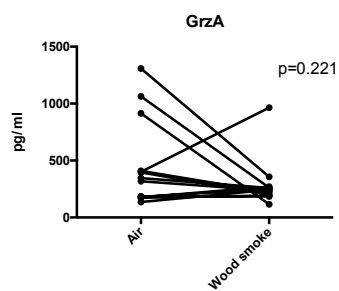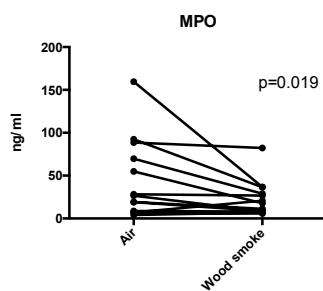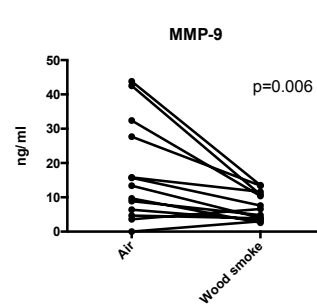

Supplement: Additional file 6: Figure S4. — Scatterplot of soluble components in bronchial wash after air and wood smoke exposure. (PDF 50 kb) [file 12989_2015_111_MOESM6_ESM.pdf]

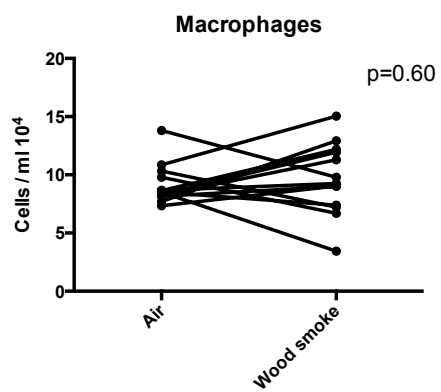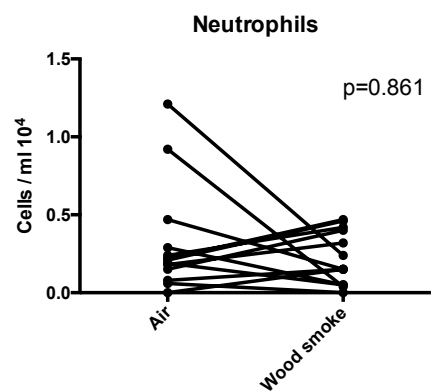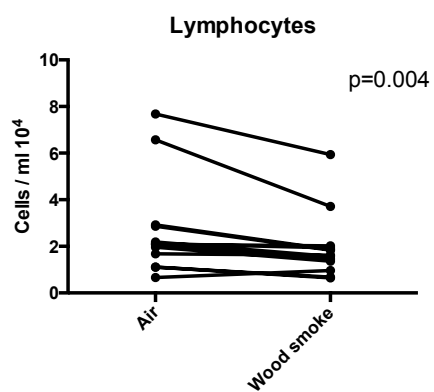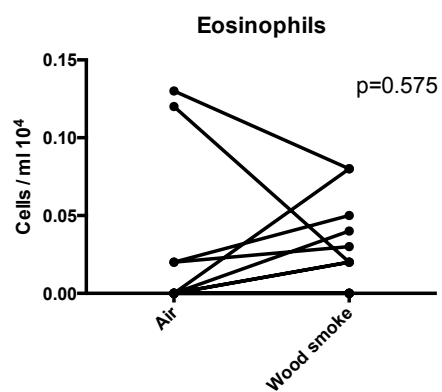

Supplement: Additional file 7: Figure S5. — Scatterplot of inflammatory cells in bronchoalveolar lavage after air and wood smoke exposure. (PDF 45 kb) [file 12989_2015_111_MOESM7_ESM.pdf]

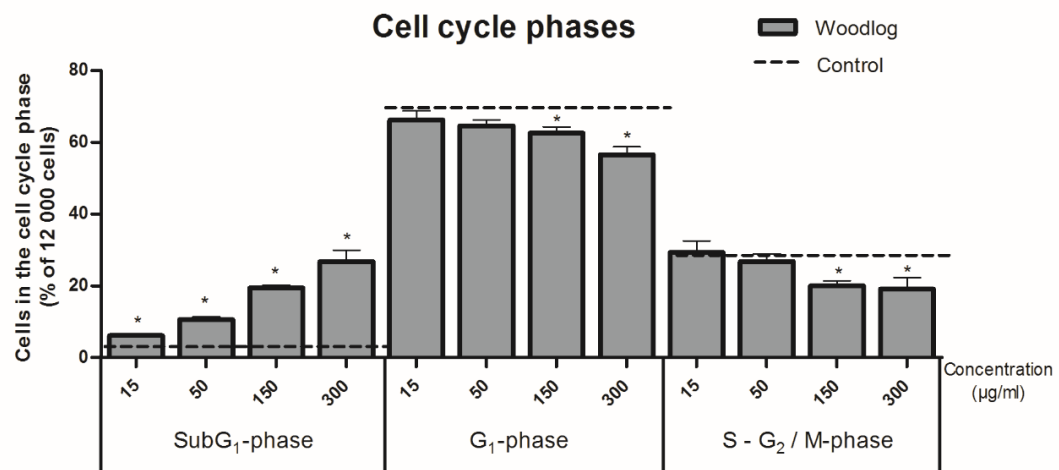

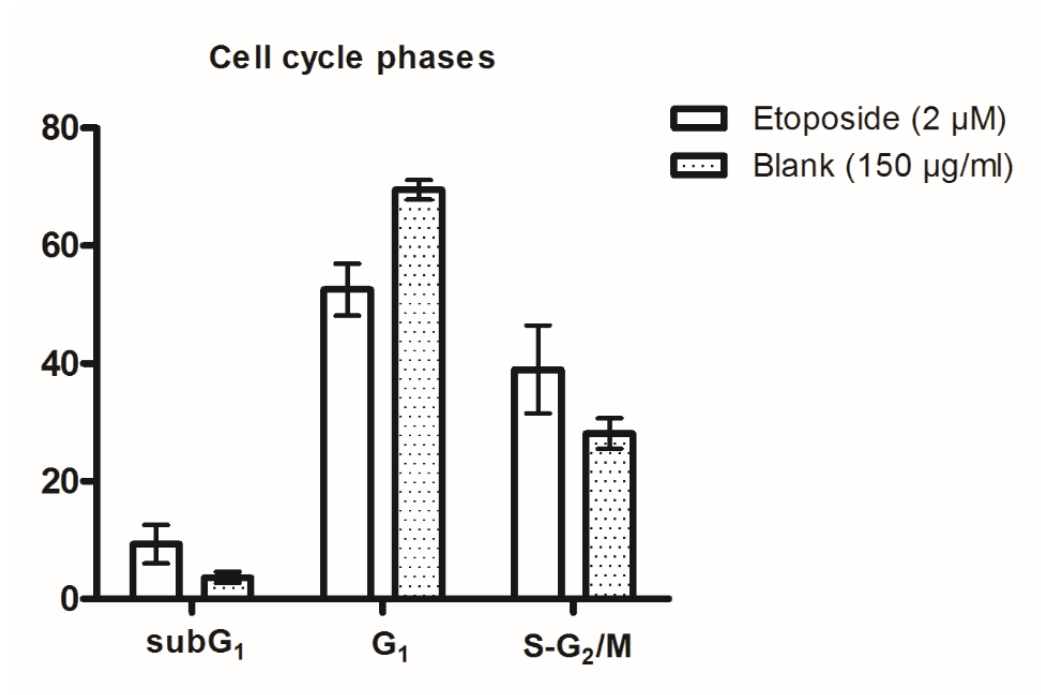

Supplement: Additional file 11: Figure S6A. — The percentages of mouse RAW264.7 macrophages in the different phases of the cell cycle (SubG1, G1 and S-G2/M) after exposure to different concentrations (15, 50, 150 and 300 μg ml-1) of emission particles from wood log combustion. Asterisks indicate statistical significance compared to control (p < 0.05) analyzed by non-parametric KruskaleWallis test. Figure S6B. The percentages of mouse RAW264.7 macrophages in the different phases of the cell cycle (SubG1, G1 and S-G2/M) after exposure to etoposide and blank substrata. (PDF 154 kb) [file 12989_2015_111_MOESM11_ESM.pdf]

**A**

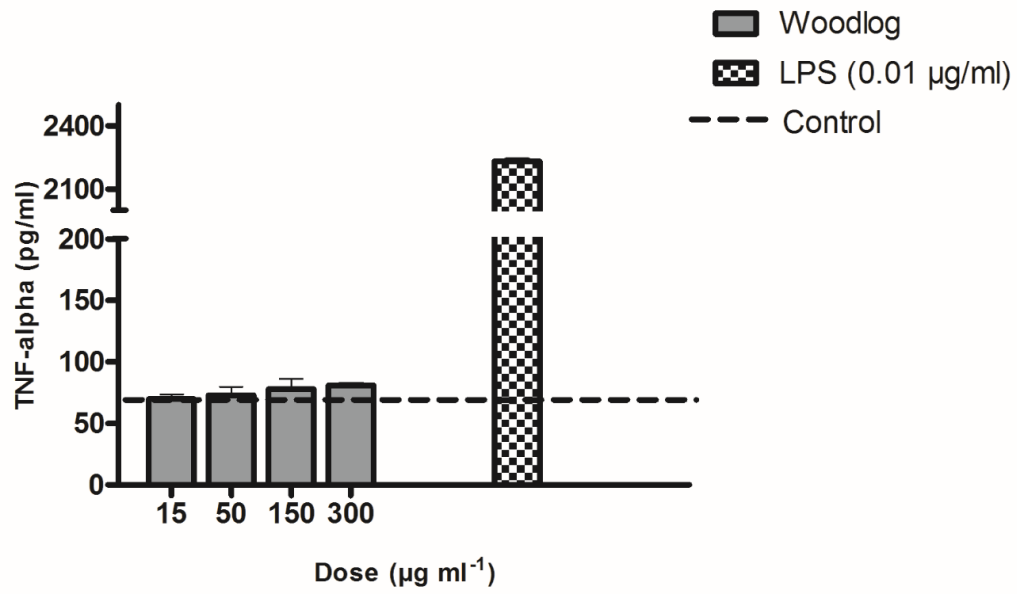

**B**

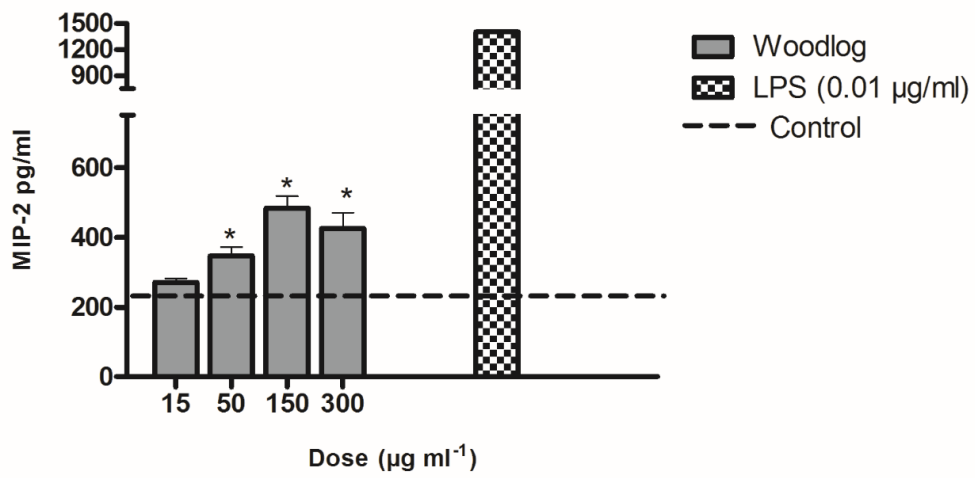

Supplement: Additional file 12: Figure S7. — Inflammation mediators tumor necrosis factor alpha (TNF-α) (A) and macrophage inflammatory protein 2 (MIP-2) (B) concentration in cell culture medium after RAW264.7 macrophages were exposure to four doses (15, 50, 150 and 300 μg ml-1) at 24 h of cell medium suspended particles emitted from a wood log combustion or lipopolysaccharide (LPS). The asterisks (*) indicate a statistically significant difference from control cells (p < 0.05, Dunnett’s test). (PDF 147 kb) [file 12989_2015_111_MOESM12_ESM.pdf]
