# Supplementary material for: Acute exposure to wood smoke from incomplete combustion - indications of cytotoxicity
Source: Part Fibre Toxicol. 2015 Oct 29;12:33. doi: 10.1186/s12989-015-0111-7 (PMC4625445; doi:10.1186/s12989-015-0111-7)
Supplement: Additional file 8: Table S3. — Soluble components in the peripheral blood from experimental exposure of 14 healthy subjects to filtered air and wood smoke. Samples were collected before (pre), at 24 h and 44 h after exposure, to air and wood smoke. Data are given as medians with interquartile range. (DOCX 62 kb) [file 12989_2015_111_MOESM8_ESM.docx]

**Table s3**: Soluble components in the peripheral blood from experimental exposure of 14 healthy subjects to filtered air and wood smoke. Samples were collected before (pre), at 24 hours and 44 hours after exposure, to air and wood smoke. Data are given as medians with interquartile range.

|  | Air  Pre 24 hrs 44 hrs | | | Wood smoke  Pre 24 hrs 44 hrs | | |
| --- | --- | --- | --- | --- | --- | --- |
| IL-6  pg/ml | 0.60  0.35-0.88 | 0.61  0.35-0.96 | 0.76  0.24-1.70 | 0.49  0.19-0.79 | 0.60  0.36-0.91 | 1.04  0.81-1.36 |
| TNF-α  pg/ml | 0.70  0.47-1.02 | 0.75  0.41-1.18 | 0.85  0.32-1.75 | 0.65  0.27-1.00 | 0.75  0.47-1.12 | 1.05  0.80-1.37 |
| s-ICAM  ng/ml | 328  284-388 | 336  268-403 | 307  362-397 | 337  274-398 | 317  285-383 | 241  287-396 |
| CC16  ng/ml | 7.00  4.52-10.87 | 5.58  4.00-9.97 | 6.30  4.82-10.02 | 5.75  3.90-10.27 | 5.90  3.07-7.57 | 5.15  4.17-12.42 |
