# Supplementary material for: Acute exposure to wood smoke from incomplete combustion - indications of cytotoxicity
Source: Part Fibre Toxicol. 2015 Oct 29;12:33. doi: 10.1186/s12989-015-0111-7 (PMC4625445; doi:10.1186/s12989-015-0111-7)
Supplement: Additional file 9: Table S4. — Lung function data from 14 subjects before (pre), immediately after (post) and 24 h after experimental exposure to filtered air and wood smoke. Data are given as mean with ± SD. (DOCX 46 kb) [file 12989_2015_111_MOESM9_ESM.docx]

**Table s4**: Lung function data from 14 subjects before (pre), immediately after (post) and 24 hours after experimental exposure to filtered air and wood smoke. Data are given as mean with ± SD.

|  | Air  Pre Post 24hrs | | | Wood smoke  Pre Post 24hrs | | |
| --- | --- | --- | --- | --- | --- | --- |
| FEV_1_ (L) | 4.01 ± 0.78 | 3.98 ± 0.78 | 4.01 ± 0.74 | 4.05 ± 0.72 | 4.04 ± 0.76 | 4.01 ± 0.73 |
| FVC (L) | 5.14 ± 1.19 | 5.11 ± 1.20 | 5.11 ± 1.22 | 5.25 ± 1.14 | 5.03 ± 1.10 | 5.07 ± 1.18 |
| FEV_1_/FVC | 0.80 ± 0.62 | 0.77 ± 0.71 | 0.78 ± 0.70 | 0.76 ± 0.72 | 0.77 ± 0.68 | 0.78 ± 0.72 |
