# Supplementary material for: Acute exposure to wood smoke from incomplete combustion - indications of cytotoxicity
Source: Part Fibre Toxicol. 2015 Oct 29;12:33. doi: 10.1186/s12989-015-0111-7 (PMC4625445; doi:10.1186/s12989-015-0111-7)
Supplement: Additional file 10: Table S5. — FENO data from 14 healthy subjects sampled before (pre), immediately after (post) and 24 h after experimental exposure to filtered air and wood smoke. Data are given as mean with ± SD. (DOCX 45 kb) [file 12989_2015_111_MOESM10_ESM.docx]

**Table s5**: FE_NO_ data from 14 healthy subjects sampled before (pre), immediately after (post) and 24 hours after experimental exposure to filtered air and wood smoke. Data are given as mean with ± SD.

|  | Air  Pre Post 24 hrs | | | Wood smoke  Pre Post 24hrs | | |
| --- | --- | --- | --- | --- | --- | --- |
| FE_NO_ 50 | 11.12 ± 5.28 | 10.12 ± 4.85 | 9.83 ±  3.39 | 9.43 ± 3.26 | 8.32 ±  3.32 | 9.08 ± 3.74 |
| FE_NO_ 10 | 33.79 ± 21.27 | 32.69 ± 17.86 | 28.30 ± 10.39 | 28.51 ± 7.72 | 26.19 ± 11.08 | 28.36 ± 9.87 |
|  | | | | | | |
